# Impact of the indigenous rotavirus vaccine Rotavac in the Universal Immunization Program in India during 2016–2020

**DOI:** 10.1038/s41591-025-03998-9

**Published:** 2025-10-07

**Authors:** Nayana P. Nair, Samarasimha N. Reddy, Sidhartha Giri, Tintu Varghese, Varunkumar Thiyagarajan, Jayaprakash Muliyil, Priya Hemavathy, Shainey Alokit Khakha, Rashmi Arora, Mohan D. Gupte, Jaqueline E. Tate, Umesh D. Parashar, Venkata Raghava Mohan, Gagandeep Kang

**Affiliations:** 1 The Wellcome Trust Research Laboratory, Christian Medical College, Vellore, India; 2 Department of Community Health, Christian Medical College, Vellore, India; 3 Translational Health Science and Technology Institute, Faridabad, India; 4 Indian Council of Medical Research, New Delhi, India; 5 Centers for Disease Control and Prevention, Atlanta, GA USA; 6 Department of Pediatrics, Kurnool Medical College and Government General Hospital, Kurnool, India; 7 Department of Pediatrics, Government General Hospital and Rangaraya Medical College, Kakinada, India; 8 Department of Pediatric Surgery, Government General Hospital and Rangaraya Medical College, Kakinada, India; 9 Department of Pediatrics, King George Hospital and Andhra Medical College, Visakhapatnam, India; 10 Department of Pediatrics, Sri Venkateswara Medical College, Tirupati, India; 11 Post Graduate Institute of Medical Sciences, Rohtak, India; 12 Shaheed Hassan Khan Mewati Government Medical College, Nalhar, India; 13 All India Institute of Medical Sciences, Raipur, India; 14 Bhagath Phool Singh Government Medical College for Women, Khanpur Kalan, Sonipat, India; 15 Department of Community Medicine and School of Public Health, Post Graduate Institute of Medical Education and Research, Chandigarh, India; 16 Department of Pediatric Surgery, Advanced Pediatric Centre, Postgraduate Institute of Medical Education and Research, Chandigarh, India; 17 Advanced Pediatric Centre, Postgraduate Institute of Medical Education and Research, Chandigarh, India; 18 Department of Radiodiagnosis and Imaging, Postgraduate Institute of Medical Education and Research, Chandigarh, India; 19 Department of Virology, Postgraduate Institute of Medical Education and Research, Chandigarh, India; 20 Indira Gandhi Medical College, Shimla, India; 21 Dr Rajendra Prasad Government Medical College, Tanda, India; 22 Department of Pediatrics, Sardar Vallabhbhai Patel Post Graduate Institute of Paediatrics, SCB MCH, Cuttack, India; 23 Department of Pediatrics, Institute of Medical Sciences and SUM Hospital, Bhubaneswar, India; 24 Department of Pediatrics, Kalinga Institute of Medical Sciences, KIIT deemed University, Bhubaneswar, India; 25 Department of Pediatrics, Hi-Tech Hospital, Bhubaneswar, India; 26 Malankara Orthodox Syrian Church Medical College Hospital, Kolencherry, India; 27 Mahatma Gandhi Memorial Medical College, Indore, India; 28 Department of Pediatrics, Sawai Man Singh Medical College, Jaipur, India; 29 Department of Microbiology, Sawai Man Singh Medical College, Jaipur, India; 30 Department of Pediatrics, Dr. Sampurnanand Medical College, Jodhpur, India; 31 Department of Pediatric Surgery, Dr. Sampurnanand Medical College, Jodhpur, India; 32 Department of Microbiology, Dr. Sampurnanand Medical College, Jodhpur, India; 33 Department of Radiology, Dr. Sampurnanand Medical College, Jodhpur, India; 34 Rabindra Nath Tagore Medical College, Udaipur, India; 35 Baptist Christian Hospital, Tezpur, India; 36 Gauhati Medical College, Guwahati, India; 37 Kanchi Kamakoti CHILDS Trust Hospital, Chennai, India; 38 ICMR-National Institute of Epidemiology, Chennai, India; 39 Institute of Child Health & hospital for Children, Chennai, India; 40 Government Rajaji Hospital and Madurai Medical College, Madurai, India; 41 King George Medical College, Lucknow, India; 42 Mangla Hospital and Research Centre, Bijnor, India; 43 Department of Pediatrics, Institute of Medical Sciences, Banaras Hindu University, Varanasi, India; 44 Baba Raghav Das Government Medical College, Gorakhpur, India

**Keywords:** Gastroenteritis, Research data

## Abstract

In 2016, India introduced Rotavac (G9P[11]), an indigenous oral rotavirus vaccine administered at 6, 10 and 14 weeks of age through the Universal Immunization Program. Evaluating its effectiveness under routine programmatic conditions is critical, given the variable performance of rotavirus vaccines in low- and middle-income countries. Here we assessed Rotavac’s real-world effectiveness and impact across 31 hospitals in 9 states between 2016 and 2020 using a test-negative case–control design. Overall, 24,624 children were enrolled in surveillance (62% male and 38% female). Of 8,372 children aged 6–59 months eligible for effectiveness analysis (1,790 rotavirus-positive cases and 5,437 rotavirus-negative controls), 6,646 received 3 doses and 581 were unvaccinated. The adjusted vaccine effectiveness of 3 doses against severe rotavirus gastroenteritis was 54% (95% confidence interval (CI) 45% to 62%), with 1,574 vaccinated cases versus 5,072 vaccinated controls. Among children aged 6–23 months (1,486 vaccinated cases and 4,595 vaccinated controls), genotype-specific adjusted vaccine effectiveness was 51% (95% CI 36% to 62%) for G3P[8], 81% (95% CI 73% to 87%) for G1P[8] and 64% (95% CI 21% to 83%) for G1P[6]. Following vaccine introduction, rotavirus positivity among hospitalized children declined from 40% to 20%. These findings confirm that Rotavac provides substantial protection against severe rotavirus disease, including nonvaccine strains, and performs comparably to internationally licensed vaccines in similar settings.

## Abstract (web summary)

In this multicentered observational study, Rotavac, an indigenous, monovalent, live, attenuated, oral rotavirus vaccine, had an adjusted vaccine efficacy of 54% and protected against severe gastroenteritis in Indian children during its rollout between 2016 and 2020.

## Main

Annually, 128,500 deaths occurring among children younger than 5 years are attributed to rotavirus, thus placing a high demand on healthcare systems^1^. Two live oral rotavirus vaccines, the monovalent human strain based Rotarix (GlaxoSmithKline Biologicals) and the pentavalent bovine-human reassortant strains containing RotaTeq (Merck and Co.), have been licensed and used internationally from 2006 onward and have successfully demonstrated a reduction in disease burden^2^. However, the vaccine performance in high mortality countries has been suboptimal^3^. Nonetheless, it has been estimated that approximately 28,900 deaths among children younger than 5 years were prevented globally in 2016 due to the use of these two vaccines^1^. Despite the availability of live-attenuated, oral rotavirus vaccines since 2006, rotavirus continues to be the most common cause of severe acute gastroenteritis (AGE) in young children in low- and middle-income countries (LMICs) with peak incidence among children aged 4–23 months^4^.

India is estimated to account for one-fifth of the global rotavirus attributed deaths, and sentinel-hospital-based surveillance during the period of 2012–2016 showed that approximately 37% gastroenteritis hospitalizations in children were due to rotavirus^5^.

In 2014, an indigenously manufactured rotavirus vaccine (Rotavac, Bharat Biotech) based on a single naturally occurring bovine-human reassortant strain, was licensed in India following clinical trials showing an efficacy of 54% against severe rotavirus gastroenteritis in children followed up for 24 months. The vaccine specifically targets group A rotavirus, which accounts for over 90% of all rotaviral gastroenteritis cases. The efficacy was comparable to that of Rotarix and RotaTeq in low-income settings^6^. The National Technical Advisory Group on Immunization (NTAGI) recommended the phased introduction of Rotavac in the Indian national vaccine program, beginning with four states, from April 2016 onward^7^.

In making their recommendations, the NTAGI emphasized the need for further monitoring of the effectiveness and impact of Rotavac. Further monitoring was suggested because vaccine performance can differ for conditions between routine programmatic use and clinical trials, especially given the variable performance of rotavirus vaccines in different populations^3^. In India, as in other LMICs, the oral polio vaccine (OPV) showed substantially diminished efficacy^8^. In addition, Rotavac is an oral, monovalent vaccine based on a unique, naturally attenuated neonatal rotavirus strain, G9P[11]. This strain has antigens that differ from those recently circulating in hospitalized Indian children; thus, examining vaccine protection against a range of strains is important^5,9^. Lastly, demonstrating the real-world impact of vaccination in reducing the burden of gastroenteritis hospitalizations provides useful evidence regarding the value of vaccination and can also help examine its potential indirect benefits in age groups not directly targeted by vaccination.

Here, we report data from a multicentric, hospital-based, observational study on the effectiveness and impact of a completed series of Rotavac against laboratory-confirmed rotavirus diarrheal admissions in Indian children. Vaccine effectiveness, assessed using a test-negative design, was stratified by age groups, nutritional status and against major circulating genotypes. Vaccine impact was assessed in a subset of hospitals with prevaccination surveillance data and compared with the postvaccination period data.

## Results

### Rotavirus vaccine effectiveness

**Fig. 1:**
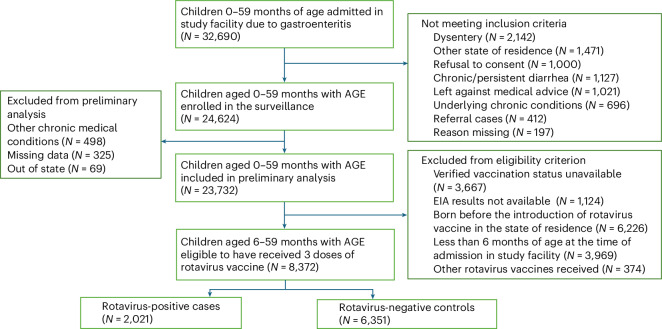
Data flow of enrolled children and identification of age-eligible children based on the date of introduction of rotavirus vaccine in the UIP. A flow diagram outlining the data collection process for study surveillance, presenting the layout of participants from initial screening to eligibility assessment and inclusion in final analysis and providing details of exclusion stepwise.

**Extended Data Fig. 1:**
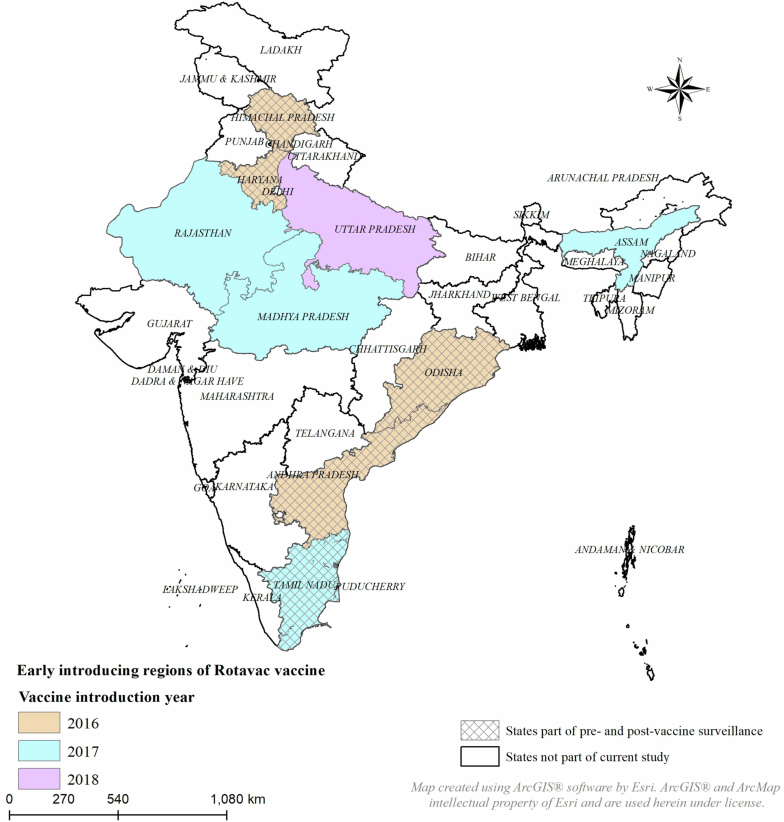
Map of India showing the phased introduction by states of Rotavac^TM^ vaccine in the Universal Immunization Program in India from 2016 to 2018. This map was generated using licensed version of ArcGIS® software to demonstrate the states in India that were part of the surveillance and introduction times of rotavirus vaccine in each of the participating states. Vaccine effectiveness surveillance data were collected from all nine states from January 2016 to January 2020. Data for assessing the impact of the vaccine were obtained from four states with pre-vaccination data from September 2012 to April 2016, post-vaccination data from May 2016 to June 2020, and from Tamil Nadu from 2018 to January 2020. A uniform protocol was followed throughout the surveillance period.

**Extended Data Fig. 2:**
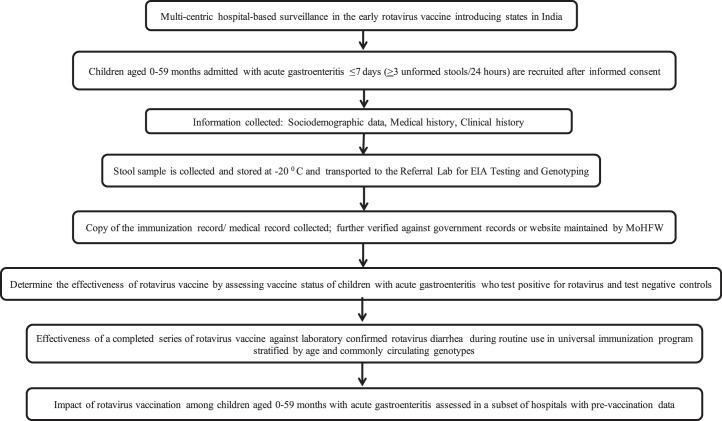
Study flow diagram. Shows the protocol followed at all the study sites for enrolment and collection of data. EIA- enzyme immunoassay; MoHFW- Ministry of Health and Family Welfare, Government of India.

**Extended Data Fig. 3:**
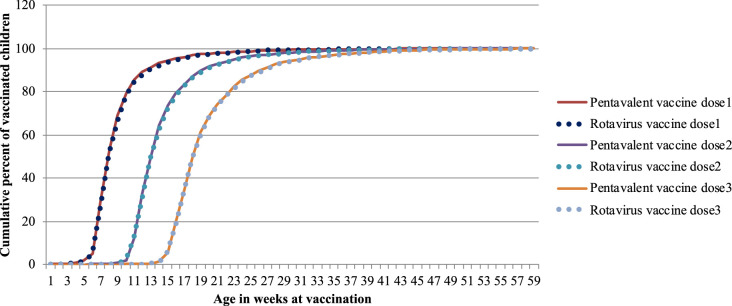
Timeliness of rotavirus and pentavalent vaccines among age-eligible children in the Universal Immunization Program in India. Rotavirus vaccines and pentavalent vaccines are administered to children at 6, 10, and 14 weeks of age in the Universal Immunization Program in India. This figure shows the comparison between cumulative percentages of vaccinated children across timelines during the surveillance. A delay is observed in the completion of the series of both vaccines.

A map of India with the details of states that were part of the surveillance are shown in Extended Data Fig. 1. Between January 2016 and January 2020, a total of 32,690 children were admitted to the surveillance network hospitals for AGE as shown in Fig. 1. The enrollment was following a detailed protocol as given in Extended Data Fig. 2. Of these, 24,624 children who fulfilled the criterion for inclusion were enrolled. Among the enrolled children, 62% were males and 38% were females. After excluding ineligible subjects, a total of 2,021 rotavirus-positive cases and 6,351 rotavirus-negative controls were included in the vaccine effectiveness analysis. Enrollment by site is described in Extended Data Table 1, and the characteristics of the cases and controls in Extended Data Table 2. The comparison of background characteristics among vaccinated and unvaccinated children is provided in Extended Data Table 3. The median age at hospital admission and sex of the cases and controls were not significantly different. This was similar among vaccinated and unvaccinated children as well. The covariates that were significantly different between the cases and controls included household size, education level of the mother, source of water supply in the household, asset score (measuring socioeconomic status), height for age *Z* (HAZ)-scores (measuring nutritional status), state of residence and maternal age. Among eligible children, positivity rates of rotavirus decreased from 50% in January to December 2016 to 23% in January 2020, and rotavirus vaccination coverage for three doses of the vaccine steadily increased from 50% in 2016 to 96% in January 2020. In Universal Immunization Program (UIP), OPV, pentavalent and rotavirus vaccine are coadministered at 6, 10, 14 weeks of age. During the study, 91%, 85%, and 77% of age-eligible children received their first, second and third doses of OPV and rotavirus vaccination on the same day (Extended Data Table 4). Extended Data Table 4 presents the difference in number of days between administration of OPV and rotavirus vaccine for the same child based on their vaccination card details. Extended Data Fig. 3 shows the difference in timeliness of an eligible child receiving each dose of pentavalent vaccine and rotavirus vaccine. A distinct time gap is observed on the basis of recommended time period of vaccine versus actually obtaining the vaccine in UIP. The time gap is more pronounced for the second and third doses of these vaccines.

In children aged 6–59 months, the adjusted vaccine effectiveness (aVE) for a completed series of Rotavac was 54% (95% confidence interval (CI) 45% to 62%), with similar vaccine effectiveness estimates in the first and second years of life (Table 1). The common circulating genotypes were G3P[8] (49%), G1P[8] (12%), G2P[4] (10%), G1P[6] (4%) and G9P[4] (3%), with 17% of rotavirus-positive samples containing mixed G and P types. Strain-specific aVE for three completed doses of the vaccine was 81% (95% CI 73% to 87%), 51% (95% CI 36% to 62%) and 32% (95% CI −23% to 62%) against G1P[8], G3P[8], and G2P[4], respectively (Table 1). The model-building approach for the unconditional logistic regression follows a stepwise selection method, incorporating both forward selection and backward elimination. Covariates were added one by one based on statistical significance, while also considering their clinical relevance. Model comparison and variable retention decisions were guided by the Bayesian Information Criterion to ensure model parsimony. Adjusting for the HAZ score, Vesikari score, site of enrollment, month and year of birth, month and year of admission, and socioeconomic status indicators did not significantly alter the effect estimates. The final analysis model does not include vaccine effectiveness based on sex of the child, as there was no significant association during preliminary analysis. Inclusion of sex variable into the final model did not improve the model strength. The final model retained covariates that were both statistically significant and contributed to the best overall goodness of fit.

**Table 1:** Effectiveness of 3 doses of rotavirus vaccine in preventing hospital admission for AGE among children aged 6–59 months, stratified by age group and genotypes

	Rotavirus-positive cases	Rotavirus-negative controls	Unadjusted vaccine effectiveness (95% CI)	aVE^a^ (95% CI)
Children aged 6–59 months	*N* = 1,790	*N* = 5,437	–	–
0 dose (reference)	216 (12%)	365 (7%)	Reference	Reference
3 doses	1,574 (88%)	5,072 (93%)	48% (38% to 57%)	54% (45% to 62%)
Stratified by age group				
Children aged 6–11 months	*N* = 887	*N* = 2697	–	–
0 dose (reference)	119 (13%)	184 (7%)	Reference	Reference
3 doses	768 (87%)	2,513 (93%)	53% (40% to 63%)	59% (47% to 68%)
Children aged 12–17 months	*N* = 601	*N* = 1,542	–	–
0 dose (reference)	54 (9%)	75 (5%)	Reference	Reference
3 doses	547 (91%)	1,467 (95%)	49% (26% to 64%)	54% (33% to 68%)
Children aged 18–23 months	*N* = 199	*N* = 675	–	–
0 dose (reference)	28 (14%)	60 (9%)	Reference	Reference
3 doses	171 (86%)	615 (91%)	41% (4% to 64%)	48% (15% to 69%)
Children aged 12–23 months	*N* = 800	*N* = 2,217	–	–
0 dose (reference)	82 (10%)	135 (6%)	Reference	Reference
3 doses	718 (90%)	2,082 (94%)	44% (25% to 58%)	51% (35% to 64%)
Children aged 6–23 months	*N* = 1,687	*N* = 4,914	–	–
0 dose (reference)	201 (12%)	319 (7%)	Reference	Reference
3 doses	1,486 (88%)	4,595 (93%)	49% (39% to 58%)	55% (45% to 63%)
Children aged 24 months and above	*N* = 103	*N* = 523	–	–
0 dose (reference)	15 (15%)	46 (9%)	Reference	Reference
3 doses	88 (85%)	477 (91%)	44% (−5% to 70%)	48% (1% to 73%)
Stratified by commonly circulating genotypes (6–23 months)				
G3P[8]	*N* = 849	*N* = 4,914	–	–
0 dose (reference)	89 (10%)	319 (7%)	Reference	Reference
3 doses	760 (90%)	4,595 (93%)	41% (25% to 54%)	51% (36% to 62%)
G1P[8]	*N* = 194	*N* = 4,914	–	–
0 dose (reference)	47 (24%)	319 (7%)	Reference	Reference
3 doses	147 (76%)	4,595 (93%)	79% (70% to 85%)	81% (73% to 87%)
G2P[4]	*N* = 162	*N* = 4,914	–	–
0 dose (reference)	13 (8%)	319 (7%)	Reference	Reference
3 doses	149 (92%)	4,595 (93%)	21% (−41% to 56%)	32% (−23% to 62%)
G1P[6]	*N* = 59	*N* = 4,914	–	–
0 dose (reference)	8 (14%)	319 (7%)	Reference	Reference
3 doses	51 (86%)	4,595 (93%)	56% (6% to 80%)	64% (21% to 83%)
G9P[4]	*N* = 52	*N* = 4,914	–	–
0 dose (reference)	7 (13%)	319 (7%)	Reference	Reference
3 doses	46 (87%)	4,595 (93%)	55% (−1% to 80%)	59% (7% to 82%)

Vaccine effectiveness of rotavirus vaccination among age-eligible children who received all 3 doses of the vaccine (*N* = 6,646) against nonvaccinated group (*N* = 581). The children not included in the table are those who received partial vaccination (*N* = 1,145). A child was considered vaccinated if the rotavirus vaccine was administered ≥14 days of hospital admission. The models that included the child’s age at the time of hospital admission, month of admission to the hospital, maternal level of education and asset score changed the adjusted odds ratio (OR) by >5% and were therefore included in the final models. A stratification of aVE by severity of AGE and dose of the vaccine is provided in Extended Data Table 5.

^a^An unconditional logistic regression was used to calculate the aVE measure.

The vaccine effectiveness estimates for the completed vaccination series among eligible children stratified by their age group and nutritional status (indicated by HAZ scores) revealed a vaccine effectiveness of 46% (95% CI 13% to 66%) among stunted children compared with that of 64% (95% CI 51% to 73%) among nonstunted children during the first year of life. The vaccine effectiveness estimates for eligible children stratified by dose of the vaccine and severity of AGE are included in Extended Data Table 5.

To check the robustness of the effectiveness estimates, we conducted a sensitivity analysis and results are included in Table 2. As the results remain stable across different models, the findings are not method dependent, and the inclusion of the *E* value suggests the strength of unmeasured confounders. The calculated *E* value is 3.77 for the main model, which shows that an unmeasured confounder would need to increase the odds of both being vaccinated and getting infected by at least 3.77× each to fully explain away the observed effect.

**Table 2:** Sensitivity analysis for aVE of 3 doses of rotavirus vaccine in preventing hospital admission for AGE among children aged 6–59 months

Type of analysis	aVE	95% CI lower limit	95% CI upper limit	Interpretation
Unmatched regression model	54%	45%	62%	Main analysis model used in the study
Matched regression model	52%	35%	64%	Values similar to main model; not adapted because of loss of sample size due to matching
Propensity score matched regression model	64%	36%	80%	Values similar to main model; not adapted because of loss of sample size due to matching
PSW regression model				
Inverse probability of treatment weights	56%	47%	63%	Among eligible children who were fully vaccinated or unvaccinated (*N* = 7,226), for estimating PSW analysis, the PS was estimated as mean PS of 0.24 and s.d. of 0.06; children in the study have 24% probability of receiving the treatment. The PS values are normally distributed in this population group. The standardized mean differences before and after weighting were assessed for covariates to see their balance between treated and control groups. Since all covariates have s.m.d. <0.1 after weighting, the model is considered well balanced
Stabilized treatment weights	56%	47%	63%	
Overlap treatment weights	48%	38%	57%	
*E* value to measure residual confounding	Measured value for OR 0.46 is 3.77			An unmeasured confounder would need to increase the odds of both being vaccinated and getting infected by at least 3.77× each to fully explain away the observed effect

A sensitivity analysis was performed to assess the aVE of 3 doses of the rotavirus vaccine in preventing hospital admissions for AGE among children aged 6–59 months (*N* = 1,790 for rotavirus-positive cases and *N* = 5,437 for rotavirus-negative controls). All models are adjusted for potential confounders, including age and socioeconomic status. The *P* values were computed using two-sided tests. PSW, propensity score weighted model; PS, propensity score; s.d., standard deviation; s.m.d., standardized mean differences.

### Rotavirus vaccine impact

**Fig. 2:**
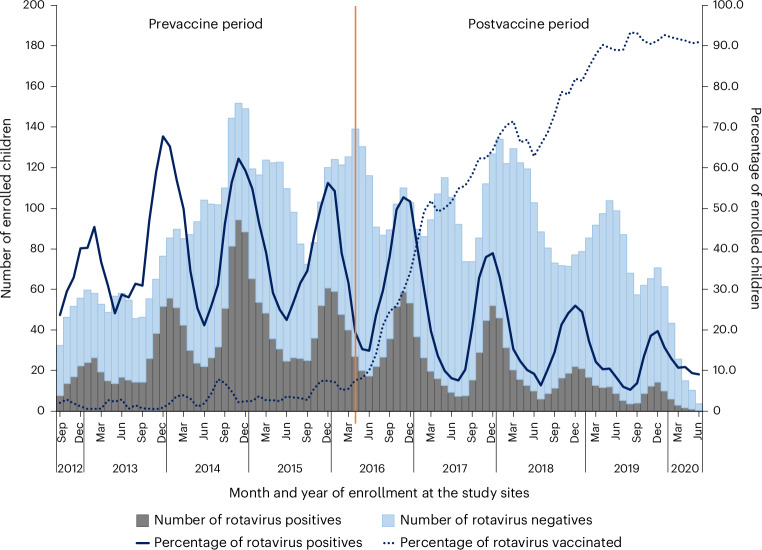
Impact of rotavirus vaccine after its introduction in the UIP in India shown by comparing the pre- and postvaccination introduction surveillance periods. A comparison of rotavirus positivity during prevaccination period and postvaccination period from five selected study sites. The bars indicate the number of rotavirus-positive cases (*N* = 2,530) and rotavirus-negative cases (*N* = 5,744) among children under 5 years admitted with AGE in the study settings by month and year of surveillance. The blue colored solid line shows the percentage of rotavirus-positives among children enrolled in the surveillance by month and year of surveillance. The blue dotted line shows the percentage of children who had received at least a single dose of rotavirus vaccine at the time of enrollment into the study surveillance. The orange straight line shows the time of rotavirus vaccine introduction into the UIP in India. The data during the prevaccine period from 2012 to 2016 were collected as a part of National Rotavirus Surveillance Network^5,35^. The postvaccine data were collected as a part of present study during the time period 2016–2020^36^.

**Fig. 3:**
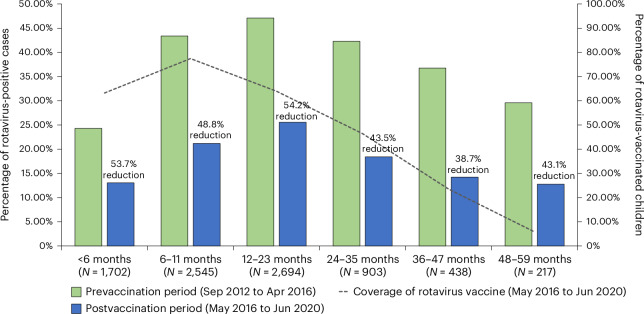
Reduction in proportions of rotaviral diarrheal disease among children under 5 years during the rotavirus vaccine postintroduction period in the study sites. The percentage reduction in rotavirus positivity among children under 5 years after the introduction of rotavirus vaccination. The green bars show the percentage of children under 5 years of age who tested positive for rotavirus gastroenteritis during the prevaccination period stratified by their age groups (*N* = 4,163). The blue bars show the percentage of children under 5 years of age who tested positive for rotavirus gastroenteritis during the postvaccination period stratified by their age groups (*N* = 4,336). The dotted line shows the coverage of rotavirus vaccine during the study period of 2016–2020 for each age group. The data during the prevaccine period were collected as a part of National Rotavirus Surveillance Network (2012–2016) using similar protocol^5,35^. Postvaccine data were collected as a part of present study (2016–2020)^36^. The details are further elaborated in Extended Data Table 6.

In the five hospitals with rotavirus surveillance data from September 2012 to June 2020, a total of 4,163 patients with AGE were enrolled from September 2012 to April 2016. Of these, 1,656 (39.8%) tested positive for rotavirus. Moreover, during the postvaccination period (May 2016 to June 2020), of a total of 4,336 children with AGE that were enrolled, 874 (21.4%) tested positive for rotavirus (Fig. 2). The rotavirus vaccination coverage that was 3.1% in September 2012 and attributable to the private sector, rose to 90.9% in June 2020. By site, declines in rotavirus detection rates were observed after vaccine introduction at each of the five sites, with progressive declines in later years with increasing vaccine coverage. The reduction in rotavirus positivity rate was 51.2% and 54.2% in infancy and among children aged 12–23 months, respectively (Fig. 3). A substantial reduction in the rate in the 24–59 month age group (ranging from 43.5% in a 24–35-month-old group to 43.1% in a 48–59-month-old group) was also noted. The vaccination coverage for these children in the postvaccination period ranged from 5.8% in the 48–59-month age group to 71.7% in the first year of life (Fig. 3). Extended Data Table 6 helps to understand the reduction rates in positivity and vaccination coverage trends across different age groups during the prevaccination and postvaccination periods and to quantify the proportion standard error and confidence limits of these estimates (Extended Data Table 6). In the interrupted time series model, the rotavirus positivity dropped by 41% showing a strong immediate effect, which is statistically significant (Table 3). The decline of rotavirus positivity as time progresses after the vaccination suggest a small but significant long-term reduction (Table 2).

**Table 3:** Interrupted time series analysis model for estimating rotavirus positivity trends among children under 5 years pre and post introduction of rotavirus vaccination

Variable	OR	95% CI	*P* value	Interpretation
Baseline positivity rate	0.77	0.68 to 0.87	1.45 × 10^−5^	Before the vaccine, the odds of positivity were 23% lower than the reference period implying mitigating effects of baseline factors
Prevaccination trend	1.0001	0.99 to 1.0003	8.24 × 10^−2^	Before vaccination, the positivity odds were stable with no significant trend
Immediate effect of vaccination	0.59	0.49 to 0.70	5.38 × 10^−9^	After vaccination, the odds of positivity dropped by 41% (a strong immediate effect)
Postvaccination trend	0.99	0.99 to 0.99	8.04 × 10^15^	After vaccination, the positivity odds remained stable (only a 1% decrease per unit of time)
Autocorrelation adjustment	–	–	–	Model accounts for autocorrelation with Newey–West standard error

An unconditional logistic regression models the probability of rotavirus positivity over time, estimating the effect of vaccine introduction by analyzing trends before and after implementation. Key adjustments include dummy variables for seasonality, correction for serial correlation, a continuous time variable for secular trends, use of rates to adjust for demographic changes, consideration of external factors and ensuring a stable baseline period. A two-sided *t*-test is used to test the null hypothesis that the trend change is zero.

## Discussion

The effectiveness of the routine use of indigenous Rotavac vaccine in the national immunization program was 54% (95% CI 45% to 62%); this was reassuringly similar to the efficacy of 54% (95% CI 35% to 67%) reported during the phase 3 vaccine trial^6^. Further, the effectiveness was sustained in the first 2 years of life, when the burden of rotavirus is greatest^1^. The effectiveness of the vaccine was high against the predominant circulating strains, G1P[8] and G3P[8], which are different G and P types from G9P[11], further emphasizing that the monovalent vaccine offers good protection against heterologous strains. By introducing the vaccine, the number of rotavirus hospitalizations was also notably reduced at the sentinel sites, with a decrease in rotavirus positivity rates to less than half of that observed in the prevaccination period (Fig. 3), further corroborating the substantial public health impact of this vaccine.

Available literature on rotavirus vaccines used in immunization programs and the importance of vaccine introduction in all high-burden settings were summarized in a review in the year 2021^2^. The performance of rotavirus vaccines since their introduction in 2006, with a focus on variations in effectiveness by socioeconomic status, age of vaccinated children and strain of rotavirus has been discussed in multiple literature reviews and meta-analyses with a recent update in the year 2022^3^. Previous studies have consistently reported that the effectiveness of vaccines made by multinational manufacturers is poorer in LMICs (53%, 95% CI 36% to 62%) than in high-income settings^10^. The effectiveness of Rotavac in India was comparable with that of other internationally licensed vaccines (Rotarix and RotaTeq) in LMICs^11–24^ (Extended Data Table 7). The effectiveness of the vaccine in preventing rotaviral AGE of any level of severity among children less than 2 years of age in Bangladesh was 41.4% (95% CI 23.2% to 55.2%), with higher effectiveness observed during the first than the later years of life (45.2%, 95% CI 26.3% to 59.3%)^25^. The waning of vaccine effectiveness during the second year of life has been reported in several studies from LMICs; however, in Africa, the waning may be variable^26^. The vaccine effectiveness estimates from Malawi 3 years after the programmatic introduction of the vaccine showed estimates of 70.6% (95% CI 33.6% to 87.0%) in the first year of life and 31.7% (95% CI −140.6% to 80.6%) in the second year^13^. A study on the effectiveness of a full series of Rotarix vaccine among children aged 4–59 months from Botswana showed a vaccine effectiveness of 54% (95% CI 23% to 73%)^4^.

When stratified by the children’s age group and nutritional status (indicated as stunted or nonstunted using the HAZ scores), the vaccine effectiveness estimates in Africa differ and the difference is more pronounced in the first than in the later years of life. Between stunted and nonstunted children, in Zimbabwe^27^, the full series of Rotarix vaccine among children aged 6–11 months showed a vaccine effectiveness of 45% versus 71%, respectively, but not in older children. In Malawi^13^, for the full series of Rotarix vaccine among eligible children, the vaccine effectiveness was 28% versus 78%, respectively. In Mozambique^28^, the vaccine effectiveness for stunted versus nonstunted children was 14% and 59%, respectively, while in Botswana^16^ and Kenya^23^, among stunted children, the vaccine provided no such protection. In our study, we noted a difference in vaccine effectiveness estimates but with overlapping confidence intervals. Before the vaccine introduction in India, Rheingans et al. had concluded that by reducing socioeconomic and geographic disparities in Indian states, the impact of rotavirus vaccine could increase, saving an additional 10,000 children’s lives each year^29^.

The effectiveness of the neonatal vaccine of G9P[11] against the major circulating strains demonstrates the heterologous protection offered by rotavirus vaccines and is consistent with studies on other rotavirus vaccines in LMICs^9,10,12–15,19,21^. The study was powered to examine strain-specific vaccine effectiveness and demonstrated effectiveness against G1P[8], G3P[8] and G2P[4]. The major currently circulating strains of rotavirus in the developing world are G1P[8], G1P[6] and G3P[8]^30^. A systematic review by Leshem et al in 2014 has shown that Rotarix and RotaTeq provide heterologous protection without changes in the circulating strain^31^. Rotavirus vaccines work less effectively in preventing severe gastroenteritis in LMICs than in more developed countries, and this phenomenon has been reported both in clinical trials and effectiveness studies^32^. The reasons for the lower effectiveness are attributed to the damaged gut environments in children in LMICs that may interfere with response to the vaccine or other as yet unknown reasons^30^. The concomitant administration of rotavirus vaccine with OPV might also suppress vaccine rotavirus multiplication in the intestinal epithelium leading to a lower vaccine response. However, high coadministration rates prevented vaccine effectiveness evaluation in children who did not receive them concomitantly^33^.

Our study showed a significant reduction in rotaviral gastroenteritis among enrolled children in the postvaccination period compared with in the prevaccination period, adding to the limited findings from other countries^2^. A decline in rotavirus positivity rate among the cases was seen in all the five prevaccination surveillance sites over time, with increasing vaccine coverage, supporting the relationship with vaccine implementation. Similar declines were not observed in nonrotavirus AGE hospitalizations. The reduction in the number of admissions due to rotavirus AGE occurred in children less than 6 months of age, suggesting that even a partial vaccine series may offer some protection. Finally, and importantly, the decline in rotavirus positivity among 2–5 year old children, many of whom were age-ineligible to receive rotavirus vaccine, exceeded the expected based on low vaccine coverage in this age group and thus suggests indirect benefits due to reduced circulation of rotavirus in the population, as have been seen in high-income countries^24^.

In the current study, we observe a risk difference of −0.14, which highlights the fact that rotavirus positivity is less likely in the group that receives rotavirus vaccination. This indicates the substantial benefit of the vaccine in reducing the disease risk by 14% in the vaccinated population. The number needed to treat to prevent one additional case of severe rotaviral gastroenteritis is 7, this emphasizes the strong impact and protective effect of the vaccine in preventing disease.

Our study has important strengths in including recruitment at multiple hospitals in nine states, a large number of enrolled children after stringent verification of vaccination data and the availability of prior surveillance data at some sites for impact assessment. Limitations include the potential for selection bias in excluding children whose vaccination status could not be verified against health registries or national data. We addressed these limitations by using the test-negative design, which avoids the selective collection of vaccine cards of cases rather than controls since the study team was unaware of the rotavirus test result for each child. The inability to verify vaccination information because of poor or missing information in government records led to the exclusion of many children. However, retention of children with unverified vaccination information may have contributed to misclassification bias, resulting in lower than actual effectiveness estimates. The test-negative case–control design may introduce selection bias because health-seeking behavior may not accurately represent the broader population, potentially leading to an overestimation or underestimation of vaccine effectiveness. In addition, this design depends on the sensitivity and specificity of the diagnostic kits used, which can result in misclassification bias. The potential for residual confounding is also considerable, which may lead to biased vaccine effectiveness estimates. Moreover, the design assumes infection with a single pathogen, which may overlook the possibility of multiple or coinfections—a common issue in low-income settings. This study seeks to mitigate these limitations through strategic planning, the use of high-quality diagnostic tools and comprehensive data collection, ensuring that the findings are both accurate and generalizable. While estimating genotype specific vaccine effectiveness estimates, the limited sample sizes for specific genotypes have led to an increase in variability in the estimates. The genetic variability of circulating rotavirus strains might also be a reason for this variability, as different strains may exhibit distinct interactions with the vaccine, leading to variation in effectiveness. This calls for additional research and the need for larger sample sizes or more focused strain surveillance, which could improve the precision of these estimates in future studies.

There are limited impact assessment studies for newly introduced vaccines in LMICs. This study demonstrates that such studies are feasible and provide real-world evidence of the impact of rotavirus vaccination. A major concern regarding introducing a new vaccine is its safety and effectiveness. We recently reported the outcomes of a safety surveillance, which demonstrated that there was no association with intussusception^34^. Our study showed that Rotavac offers good protection against severe rotaviral gastroenteritis, including against genotypes excluded from the vaccine, in Indian children. Pre- and postintroduction data demonstrated a reduction in the overall rotavirus positivity rate in children hospitalized for gastroenteritis and in children not age-eligible for the vaccine, indicating an important public health impact. This affordable vaccine, which has been recently prequalified, is being rolled out in the Global Alliance for Vaccines and Immunization-eligible countries and will contribute to reducing global rotavirus burden.

## Methods

### Ethical approval

Ethical approval was obtained from Institutional Ethics Committee of Christian Medical College, Vellore, as well as from the institutional review boards of all participating hospitals. The ethics committees of all participating institutions approved the study as detailed in the published protocol^36^. Written informed consent was obtained from the parent or legal guardian of each enrolled child before data and sample collection. Any child who satisfied the study protocol were enrolled into the surveillance. All data were anonymized before analysis to ensure participant confidentiality. The 31 participating institutions are: KMCGGH, Kurnool, Andhra Pradesh, GGHRMC, Kakinada, Andhra Pradesh, KGHAMC, Vishakhapatnam, Andhra Pradesh, SVMC, Tirupati, Andhra Pradesh, RPGMC, Tanda, Himachal Pradesh, IGMC, Shimla, Himachal Pradesh, PGIMS, Rohtak, Haryana, SHKMGMC, Mewat, Haryana, BPSGMCW, Sonipat, Haryana, PGIMER, Chandigarh, Haryana, SVBPPGIP, Cuttack, Odisha, IMS, SUM, Bhubaneswar, Odisha, KIMS, Bhubaneswar, Odisha, Hi-Tech, Bhubaneswar, Odisha, MGMMC, Indore, Madhya Pradesh, SMSMC, Jaipur, Rajasthan, RNTMC, Udaipur, Rajasthan, SNMC, Jodhpur, Rajasthan, KKCTH, Chennai, Tamil Nadu, ICH, Chennai, Tamil Nadu, Christian Medical College (CMC), Vellore, Tamil Nadu, GVMC, Vellore, Tamil Nadu, Narayani, Vellore, Tamil Nadu, Nalam hospital, Vellore, Tamil Nadu, GRHMMC, Madurai, Tamil Nadu, BCH, Tezpur, Assam, GMC, Guwahati, Assam, KGMU, Lucknow, Uttar Pradesh, Mangla Hospital, Bijnor, Uttar Pradesh, IMS, BHU, Varanasi, Uttar Pradesh, BRDGMC, Gorakhpur, Uttar Pradesh.

### Ethics and inclusion statement

Researchers from all 31 participating institutions were actively involved in the study design and responsible for obtaining ethical clearance from their respective institutional ethics committees, as well as implementing the study at their sites. Data ownership remains with the principal investigator at each institution, as outlined in formal memorandums of understanding, which were strictly adhered to. Many of the site investigators have previously published site-specific data to better characterize regional disease burden. All researchers from participating institutions are recognized as collaborators in the Rotavirus Vaccine Effectiveness and Impact Assessment Network and are included as coauthors of this paper. The data generated are unique to each site and offer valuable insights into the geographic variability in rotavirus disease burden across India. A supplement dedicated to this work was published in the Indian Journal of Pediatrics. The memorandums of understanding established before study initiation clearly defined the roles and responsibilities of each collaborator and, where feasible, included plans for capacity-building at participating institutions. No exceptions to standard ethical requirements were made, and approvals were obtained from each institution’s local ethics committee. As this was an observational study, regulations related to animal welfare, environmental protection and biorisk were not applicable. The study posed no risk of stigmatization, discrimination or personal harm to participants, and informed consent was obtained from parents or caregivers of all enrolled children. This research did not involve any transfer of biological materials, cultural artifacts or traditional knowledge outside the country. Relevant local and regional research has been appropriately cited. No health, safety or security risks to researchers were involved in conducting this study.

### Study design

From January 2016 to January 2020, a prospective, active surveillance for rotavirus gastroenteritis was conducted in a network of 31 sentinel hospitals in rural and urban areas of the first nine Indian states to introduce Rotavac (Extended Data Fig. 1). This observational study uses long-term surveillance data from states that introduced rotavirus vaccine to estimate vaccine effectiveness using test negative case control study design and impact of the vaccine is assessed by comparing the rotavirus-positive and rotavirus-negative cases during the pre- and postvaccination periods. Details of the study are described in a published protocol^36^. A study flow diagram is shown in Extended Data Fig. 2. Briefly, information on all children admitted for gastroenteritis in each sentinel hospital was recorded in an admission logbook, with those aged 0–59 months eligible for this study. Rotaviral diarrhea distinctly exhibits a sex difference, often the ratio being 4:1 for males and females respectively in resource poor settings. Sex of the child plays a key role in analysis and results reported accordingly. The sex of the child enrolled in the study was determined on the basis of biological attribute. The sex of the child was recorded by the study personnel after examination of the child.

A detailed clinical history, sociodemographic information and vaccination history were collected. A stool sample of 5 ml was collected in a sterile screw-capped container and stored at each site at −20 °C until cold chain transported to the Wellcome Trust Research Laboratory, CMC, Vellore. The samples are tested using the Premier Rotaclone, (Meridian Biosciences) commercially available enzyme immunoassay (EIA). According to the manufacturer’s kit insert, the EIA kit used in the study, Premier Rotaclone, (Meridian Biosciences), has a sensitivity of >99% and specificity of 92–97%. However, two other studies have found the Premier Rotaclone to have a sensitivity of 76.8–80.7% and specificity of 100% (refs. ^37,38^). All EIA-positive samples were genotyped using published methods^39,40^. All the samples were genotyped using the WHO generic protocol for rotavirus surveillance. For EIA-positive samples that could not be genotyped, VP6 PCR was used to confirm for rotavirus positivity. A Sanger sequencing method was used to sequence the untyped samples and unusual rotavirus strains^9,41^.

### Data management

Clinical and sociodemographic data captured on paper case report forms were collected from each site and sent periodically to CMC, Vellore, where the data were checked and entered into the Structured Query Language software database. The data were analyzed using licensed software’s Stata v.14.2 (StataCorp LLC) and Microsoft Excel v.16.78.

### Statistical analysis

#### Rotavirus vaccine effectiveness

We used the test-negative case–control study design to estimate vaccine effectiveness in age-eligible children. The cases were defined as children with AGE who tested positive for rotavirus infection using EIA, whereas the controls had tested negative^42^. To define age-eligibility for rotavirus vaccination, only children born 6 weeks before the vaccine introduction date by state or after vaccine introduction were considered eligible. Because our objective was to determine the effectiveness of a full three-dose series of Rotavac, we restricted the vaccine effectiveness analysis to children 6 months of age and older given delays in vaccine dose administration in the Universal Immunization Program in India. Although, the three rotavirus vaccine doses are recommended at 6, 10 and 14 weeks, approximately 90% of eligible and vaccinated children in our study received all three doses of the vaccine by 27 weeks of age (Extended Data Fig. 3). A child was considered vaccinated with a particular dose if the vaccine was given 14 days before admission to the study hospital. The vaccination status of children was determined from a copy of the parent-retained immunization record. Incomplete or unavailable information was also recorded and confirmed with the government held immunization records at either the Health Sub-Centre, Primary Health Centre or the immunization website managed by the Ministry of Health and Family Welfare. Where confirmation of vaccination status was not possible, the child was excluded from the analysis.

To assess a vaccine effectiveness of 40%, we estimated that a minimum of 242 cases of rotavirus AGE were required with a control-to-case ratio of 2:1 and an expected vaccine coverage of 80% among controls^36^. Additional cases were enrolled to address the secondary objectives specified in the protocol^36^. Unconditional logistic regression was used to estimate odds ratios (OR) and 95% CIs. We calculated vaccine effectiveness as follows:

1 $$\mathrm{VE}=1-\mathrm{OR}.$$

The OR was obtained from the regression models. The models built for estimating aVE included the admission hospital, educational status of the mother, assets owned by the family and age of the child in days at the time of hospital admission. Covariates were included in the model if their inclusion changed the OR associated with vaccination by more than 5%. Sex, birth month, birth year, year of hospital admission, hospital of admission and household characteristics were excluded from the final model. Stratified analyses were performed to assess aVE by age, nutritional status, state of residence, severity of diarrhea, dose of the vaccine and by circulating genotypes. An interaction term between vaccination status (completely vaccinated/unvaccinated) and age in months was included in the main model to assess the statistical significance of the resulting vaccine effectiveness. The full model (with the interaction term) was compared with a reduced model (without the interaction term) using a likelihood ratio test. The resulting *P* value from the likelihood ratio test was used to evaluate model fit. Inclusion of the interaction term of vaccination status and age of the child was not statistically significant and excluded from the final model. All reported aVE estimates were against admission to the hospital for acute rotavirus diarrhea.

To address potential confounding effects, we conducted a sensitivity analysis using various statistical techniques. The methods include an unmatched logistic regression, as adapted in the current study; matched regression analysis, where participants were matched based on relevant covariates before regression; propensity score matched regression, which matches participants based on propensity scores to reduce confounding; propensity score weighted regression, which weights observations based on propensity scores to balance covariates between the vaccine and nonvaccine groups; and estimation of the *E* value, which calculates the minimum strength of an unmeasured confounder needed to nullify the observed association. Estimation of the *E* value to measure residual confounding in the regression model was done using the formula

2 $$E=1/\mathrm{OR}+{\rm{\surd }}1/\mathrm{OR}(1/\mathrm{OR}-1).$$

Comparing the main effectiveness estimates across these methods allows us to assess the stability of our findings and validate the statistical approach used in this study^43,44^.

#### Rotavirus vaccine impact

Of the 31 sentinel hospitals, 5 had conducted rotavirus surveillance as part of the National Rotavirus Surveillance Network from 2012 onward, based on a protocol similar to that in this study^5,39,40^. Thus, the assessment of the impact of the vaccine in the states of Andhra Pradesh, Himachal Pradesh, Haryana, Odisha and Tamil Nadu was enabled (Extended Data Fig. 1). We compared the rotavirus-positive and rotavirus-negative cases during the pre- and postvaccination periods. The age distribution and reduction in rates of rotavirus positivity before and after vaccine introduction were also analyzed. An interrupted time series analysis was conducted to demonstrate the effects of vaccine independently from pre-existing trends or other external influences^45^. This approach helps evaluate both the immediate and long-term impact of vaccine introduction.

### Reporting summary

Further information on research design is available in the Nature Portfolio Reporting Summary linked to this article.

## Online content

Any methods, additional references, Nature Portfolio reporting summaries, source data, extended data, supplementary information, acknowledgements, peer review information; details of author contributions and competing interests; and statements of data and code availability are available at 10.1038/s41591-025-03998-9.

## Supplementary information

Reporting Summary

## Figures and Tables

**Extended Data Table 1:**
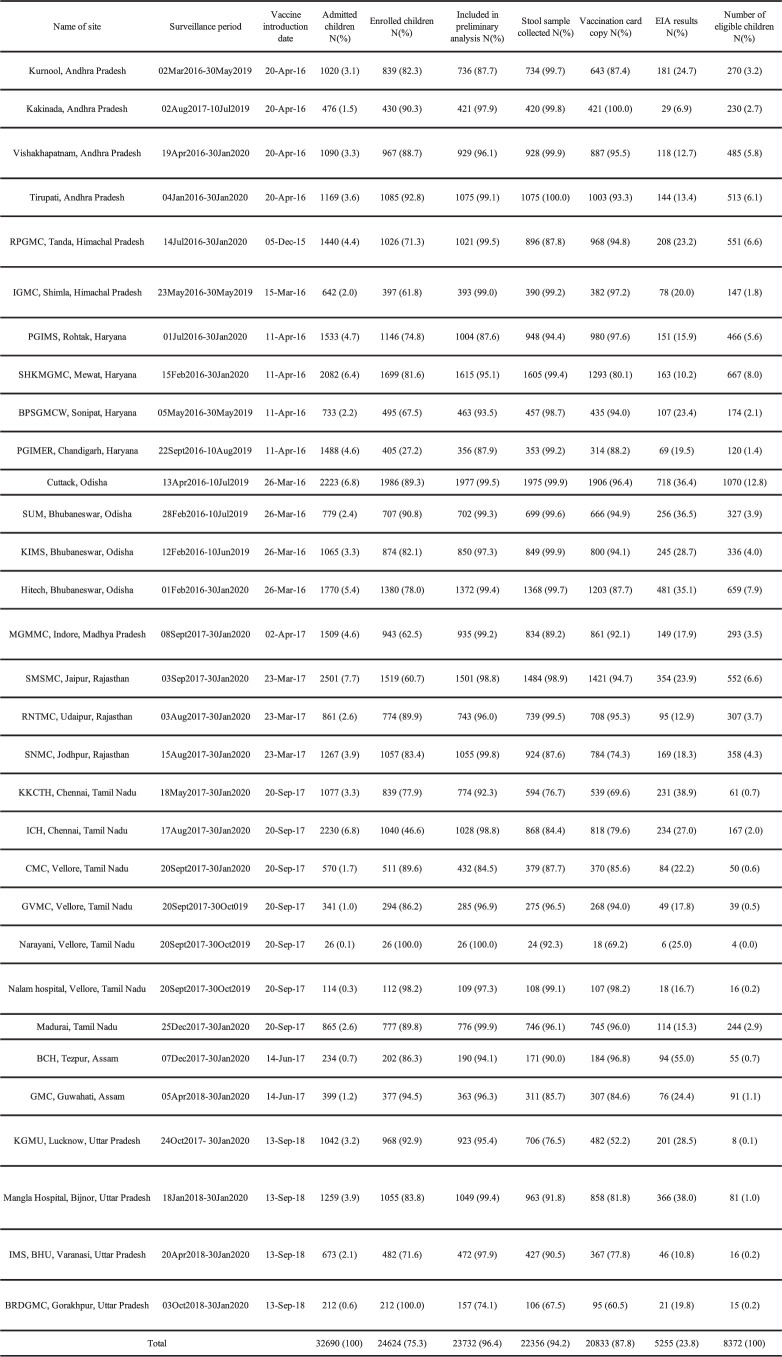
Admission and enrolment of acute gastroenteritis cases among under-five children in the surveillance in the study sites

This table provides details of the institutions that participated in the surveillance including start and end dates, number of children screened for the surveillance and further enrolled into the study and preliminary data collected for the surveillance sorted by each study site. EIA- enzyme immunoassay.

**Extended Data Table 2:**
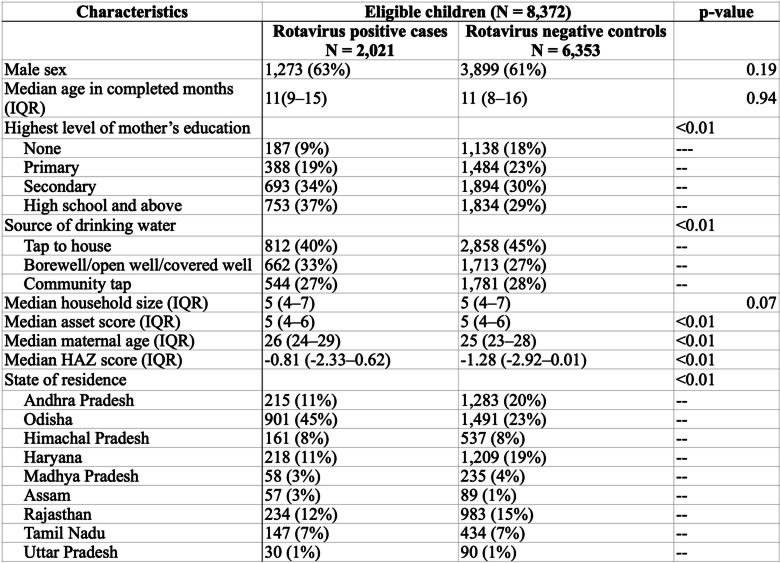
Baseline characteristics of eligible children

All continuous variables were not normally distributed and were therefore assessed using the Wilcoxon rank-sum test; p < 0.05 was considered significant. The χ2 test was used to assess dichotomous and categorical variables; p < 0.05 was considered significant. For each characteristic, the total and missing value are excluded in the respective bivariate analysis.

IQR - interquartile range; HAZ- height for age Z-score.

**Extended Data Table 3:**
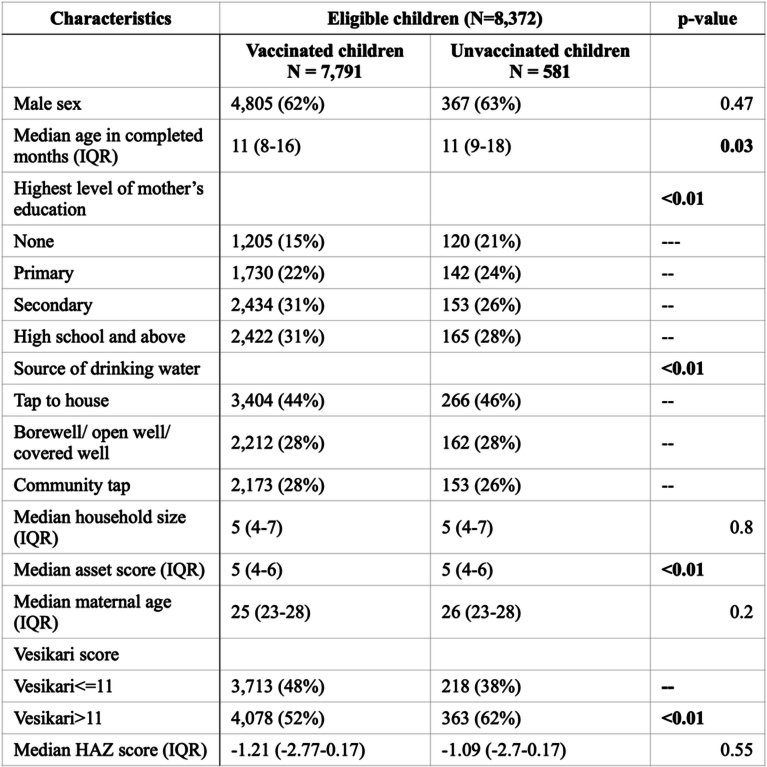
Comparison of baseline characteristics among rotavirus vaccinated and rotavirus unvaccinated children

All continuous variables were not normally distributed and were therefore assessed using the Wilcoxon rank-sum test; p < 0.05 was considered significant. The χ2 test was used to assess dichotomous and categorical variables; p < 0.05 was considered significant. For each characteristic, the total and missing value are excluded in the respective bivariate analysis.

IQR- interquartile range; HAZ- height for age Z-score.

**Extended Data Table 4:**
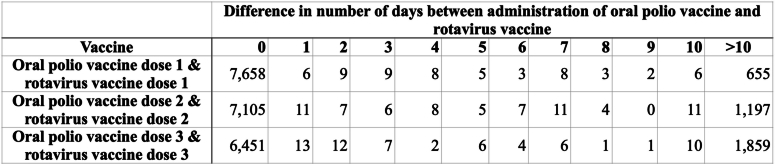
Administration of oral polio vaccine and rotavirus vaccine among eligible children in the surveillance

In India, the rotavirus vaccine is administered along with oral polio vaccine (OPV) at 6, 10, and 14 weeks of age. This table shows the day when OPV and rotavirus vaccine was administered to age-eligible children per the dose of the vaccine according to the immunization records. As per these data, 91% of children received the first dose of OPV and rotavirus vaccine on the same day, 85% received a second dose of both vaccines on the same day and 77% received a third dose of both vaccines on the same day.

**Extended Data Table 5:**
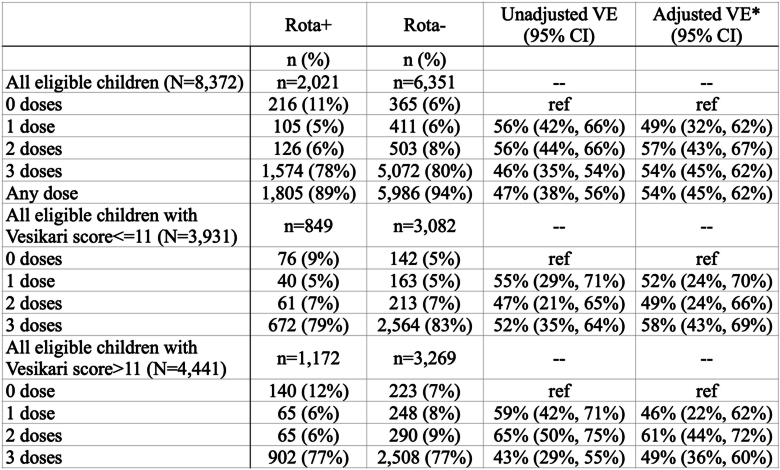
Effectiveness of rotavirus vaccine in preventing hospital admission for acute gastroenteritis among children aged 6–59 months, stratified by dose response and severity of gastroenteritis

A child is considered vaccinated if the rotavirus vaccine was administered >=14 days at the time of hospital admission. *Unconditional logistic regression used to calculate adjusted vaccine effectiveness. Models that included age of the child in months at the time of hospital admission and maternal education (categorical) changed the adjusted odds ratio by >5% and were therefore included in the final model. Sex, birth month, birth year, month of admission, year of admission, socioeconomic variables and variables of household characteristics were not included in the final model.

**Extended Data Table 6:**
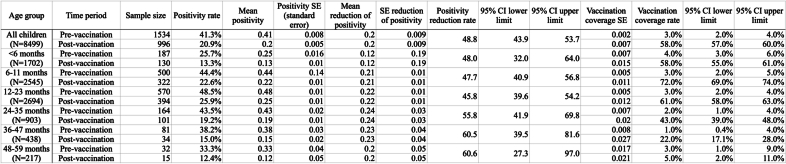
Proportion of rotavirus positives before and after the vaccine introduction, stratified by age groups

This table helps to understand the reduction rates in positivity and vaccination coverage trends across different age groups during the pre- and post-vaccination periods, and to quantify the proportion, standard error (SE), and confidence limits of these estimates.

SE- Standard Error; CI- Confidence Interval.

**Extended Data Table 7:**
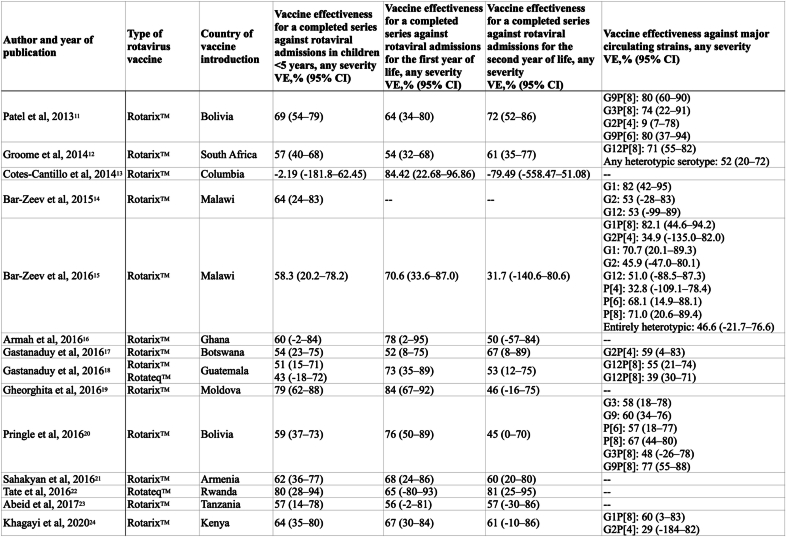
Comparison of vaccine effectiveness estimates for internationally licensed rotavirus vaccines in low- middle-income countries using the test-negative case-control design

This table provides a clear view of existing literature and readers can easily comprehend the published works from different low-middle-income countries that have introduced a rotavirus vaccine using test negative case-control study design.

VE- vaccine effectiveness; CI -confidence interval.

## Data Availability

The data used in this study were collected through hospital-based surveillance conducted in early Rotavac-introducing regions of India between 2016 and 2020, using a uniform protocol previously published by the authors. The study includes newly generated data to assess vaccine effectiveness post introduction, as well as pre- and postvaccination data from five hospitals to evaluate vaccine impact. The data during prevaccination period of 2012–2016 were collected as a part of National Rotavirus Surveillance Network using similar protocol. While the corresponding authors retain ownership of the data, portions have been previously published as part of the broader surveillance network^5,35^. All the data generated and/or analyzed for the findings of this study are openly available via GitHub at https://github.com/drvenkatm/Rotavac_VE_India.git. The dataset includes all relevant variables used in the analysis and is accessible for public use.

## Code availability

The code used for data analysis, model estimation and results generation is available via GitHub at https://github.com/drvenkatm/Rotavac_VE_India.git. The repository contains detailed instructions for reproducing the analysis, including dependencies and setup guidelines. All scripts are provided for open use and further research.
