# Author Correction: Impact of the indigenous rotavirus vaccine Rotavac in the Universal Immunization Program in India during 2016–2020

**DOI:** 10.1038/s41591-025-04062-2

**Published:** 2025-10-27

**Authors:** Nayana P. Nair, Samarasimha N. Reddy, Sidhartha Giri, Tintu Varghese, Varunkumar Thiyagarajan, Jayaprakash Muliyil, Priya Hemavathy, Shainey Alokit Khakha, Rashmi Arora, Mohan D. Gupte, Jaqueline E. Tate, Umesh D. Parashar, Venkata Raghava Mohan, Gagandeep Kang

**Affiliations:** 1 The Wellcome Trust Research Laboratory, Christian Medical College, Vellore, India; 2 Department of Community Health, Christian Medical College, Vellore, India; 3 Translational Health Science and Technology Institute, Faridabad, India; 4 Indian Council of Medical Research, New Delhi, India; 5 Centers for Disease Control and Prevention, Atlanta, GA USA; 6 Department of Pediatrics, Kurnool Medical College and Government General Hospital, Kurnool, India; 7 Department of Pediatrics, Government General Hospital and Rangaraya Medical College, Kakinada, India; 8 Department of Pediatric Surgery, Government General Hospital and Rangaraya Medical College, Kakinada, India; 9 Department of Pediatrics, King George Hospital and Andhra Medical College, Visakhapatnam, India; 10 Department of Pediatrics, Sri Venkateswara Medical College, Tirupati, India; 11 Post Graduate Institute of Medical Sciences, Rohtak, India; 12 Shaheed Hassan Khan Mewati Government Medical College, Nalhar, India; 13 All India Institute of Medical Sciences, Raipur, India; 14 Bhagath Phool Singh Government Medical College for Women, Khanpur Kalan, Sonipat, India; 15 Department of Community Medicine and School of Public Health, Post Graduate Institute of Medical Education and Research, Chandigarh, India; 16 Department of Pediatric Surgery, Advanced Pediatric Centre, Postgraduate Institute of Medical Education and Research, Chandigarh, India; 17 Advanced Pediatric Centre, Postgraduate Institute of Medical Education and Research, Chandigarh, India; 18 Department of Radiodiagnosis and Imaging, Postgraduate Institute of Medical Education and Research, Chandigarh, India; 19 Department of Virology, Postgraduate Institute of Medical Education and Research, Chandigarh, India; 20 Indira Gandhi Medical College, Shimla, India; 21 Dr Rajendra Prasad Government Medical College, Tanda, India; 22 Department of Pediatrics, Sardar Vallabhbhai Patel Post Graduate Institute of Paediatrics, SCB MCH, Cuttack, India; 23 Department of Pediatrics, Institute of Medical Sciences and SUM Hospital, Bhubaneswar, India; 24 Department of Pediatrics, Kalinga Institute of Medical Sciences, KIIT deemed University, Bhubaneswar, India; 25 Department of Pediatrics, Hi-Tech Hospital, Bhubaneswar, India; 26 Malankara Orthodox Syrian Church Medical College Hospital, Kolencherry, India; 27 Mahatma Gandhi Memorial Medical College, Indore, India; 28 Department of Pediatrics, Sawai Man Singh Medical College, Jaipur, India; 29 Department of Microbiology, Sawai Man Singh Medical College, Jaipur, India; 30 Department of Pediatrics, Dr. Sampurnanand Medical College, Jodhpur, India; 31 Department of Pediatric Surgery, Dr. Sampurnanand Medical College, Jodhpur, India; 32 Department of Microbiology, Dr. Sampurnanand Medical College, Jodhpur, India; 33 Department of Radiology, Dr. Sampurnanand Medical College, Jodhpur, India; 34 Rabindra Nath Tagore Medical College, Udaipur, India; 35 Baptist Christian Hospital, Tezpur, India; 36 Gauhati Medical College, Guwahati, India; 37 Kanchi Kamakoti CHILDS Trust Hospital, Chennai, India; 38 ICMR-National Institute of Epidemiology, Chennai, India; 39 Institute of Child Health & hospital for Children, Chennai, India; 40 Government Rajaji Hospital and Madurai Medical College, Madurai, India; 41 King George Medical College, Lucknow, India; 42 Mangla Hospital and Research Centre, Bijnor, India; 43 Department of Pediatrics, Institute of Medical Sciences, Banaras Hindu University, Varanasi, India; 44 Baba Raghav Das Government Medical College, Gorakhpur, India

**Keywords:** Gastroenteritis, Research data

Correction to: *Nature Medicine* 10.1038/s41591-025-03998-9, published online 7 October 2025.

In the version of the article initially published, Tintu Varghese (The Wellcome Trust Research Laboratory, Christian Medical College, Vellore, India) was missing from the author list and is now included, while the affiliation numbers shown for some Collaborators of the rotavirus vaccine effectiveness and impact assessment network members were incorrect. The author list and affiliations are now updated in the HTML and PDF versions of the article.

