# Supplementary material for: Impact of the indigenous rotavirus vaccine Rotavac in the Universal Immunization Program in India during 2016–2020
Source: Nat Med. 2025 Oct 7;31(11):3871–8. doi: 10.1038/s41591-025-03998-9 (PMC12618247; doi:10.1038/s41591-025-03998-9)
Supplement: Supplementary file 1 — Reporting Summary [file 41591_2025_3998_MOESM1_ESM.pdf]

nature portfolio

Corresponding author(s): Venkata Raghava Mohan

Last updated by author(s): Jun 28, 2025

Reporting Summary

Nature Portfolio wishes to improve the reproducibility of the work that we publish. This form provides structure for consistency and transparency in reporting. For further information on Nature Portfolio policies, see our Editorial Policies and the Editorial Policy Checklist.

Statistics

For all statistical analyses, confirm that the following items are present in the figure legend, table legend, main text, or Methods section.

n/a	Confirmed	
☐	☒	The exact sample size (*n*) for each experimental group/condition, given as a discrete number and unit of measurement
☐	☒	A statement on whether measurements were taken from distinct samples or whether the same sample was measured repeatedly
☐	☒	The statistical test(s) used AND whether they are one- or two-sided<br>*Only common tests should be described solely by name; describe more complex techniques in the Methods section.*
☐	☒	A description of all covariates tested
☐	☒	A description of any assumptions or corrections, such as tests of normality and adjustment for multiple comparisons
☐	☒	A full description of the statistical parameters including central tendency (e.g. means) or other basic estimates (e.g. regression coefficient) AND variation (e.g. standard deviation) or associated estimates of uncertainty (e.g. confidence intervals)
☐	☒	For null hypothesis testing, the test statistic (e.g. *F*, *t*, *r*) with confidence intervals, effect sizes, degrees of freedom and *P* value noted<br>*Give P values as exact values whenever suitable.*
☒	☐	For Bayesian analysis, information on the choice of priors and Markov chain Monte Carlo settings
☒	☐	For hierarchical and complex designs, identification of the appropriate level for tests and full reporting of outcomes
☒	☐	Estimates of effect sizes (e.g. Cohen's *d*, Pearson's *r*), indicating how they were calculated

Our web collection on statistics for biologists contains articles on many of the points above.

Software and code

Policy information about availability of computer code

Data collection	Data was collected from under five children who were hospitalized in the study setting due to an episode of acute gastroenteritis after obtaining informed consent from primary caregivers. Data was collected in paper forms and collated at the Central Institution where data was entered using the software Structured Query Language (SQL) and any queries generated were clarified with respective institutions within a recognized time frame.
Data analysis	The data were analyzed using licensed software's Stata v.14.2 (StataCorp LLC) and Microsoft® Excel v.16.78. The dataset as well as codes available for public via GitHub repository.

For manuscripts utilizing custom algorithms or software that are central to the research but not yet described in published literature, software must be made available to editors and reviewers. We strongly encourage code deposition in a community repository (e.g. GitHub). See the Nature Portfolio guidelines for submitting code & software for further information.

Data

Policy information about availability of data

All manuscripts must include a data availability statement. This statement should provide the following information, where applicable:

- Accession codes, unique identifiers, or web links for publicly available datasets
- A description of any restrictions on data availability
- For clinical datasets or third party data, please ensure that the statement adheres to our policy

The data generated and/or analyzed for the findings of this study are openly available in GitHub at https://github.com/drvenkatm/Rotavac_VE_India.git. The dataset includes all relevant variables used in the analysis and is accessible for public use, subject to the terms outlined in the repository.

Research involving human participants, their data, or biological material

Policy information about studies with human participants or human data. See also policy information about sex, gender (identity/presentation), and sexual orientation and race, ethnicity and racism.

Reporting on sex and gender	Sex based analysis was done in this study. Based on existing literature, rotaviral diarrhea distinctly exhibits a sex difference, often the ratio being 4:1 for males and females respectively in resource poor settings. Sex of the child plays a key role in analysis and results reported accordingly. The sex of the child enrolled in the study was determined on the basis of biological attribute. The sex of the child was observed by the study personnel during examination and sample collection. In this study, among the children enrolled into the surveillance, 62% were males and 38% were females. The same is applicable for children included in the final case control evaluation as well. The final analysis model does not include vaccine effectiveness based on sex of the child as there was no significant association during preliminary analysis. inclusion of sex variable into the final model did not improve the model strength.
Reporting on race, ethnicity, or other socially relevant groupings	Data was collected from hospitals and care was taken to include all eligible children. When looking into background characteristics of enrolled children, such differences were uniformly distributed across different groups under study. Supplementary Tables 2&3 gives background characteristics of children included in final analysis.
Population characteristics	Population characteristics described in Supplementary Tables 2 & 3
Recruitment	Recruitment was from hospital settings identified prior to the start of the study
Ethics oversight	Ethical clearance was obtained from Institutional Review Board of Christian Medical College, Vellore, Tamil Nadu, India and also from the ethics committees of each of the 31 participating institutions, details of which are already included as part of protocol published for this study. The 31 participating institutions are: KMCGGH, Kurnool, Andhra Pradesh, GGHRMC, Kakinada, Andhra Pradesh, KGHAMC, Vishakhapatnam, Andhra Pradesh, SVMC, Tirupati, Andhra Pradesh, RPGMC, Tanda, Himachal Pradesh, IGMC, Shimla, Himachal Pradesh, PGIMS, Rohtak, Haryana, SHKMGMC, Mewat, Haryana, BPSGMCW, Sonipat, Haryana, PGIMER, Chandigarh, Haryana, SVBPPGIP, Cuttack, Odisha, IMS, SUM, Bhubaneswar, Odisha, KIMS, Bhubaneswar, Odisha, Hi-Tech, Bhubaneswar, Odisha, MGMMC, Indore, Madhya Pradesh, SMSMC, Jaipur, Rajasthan, RNTMC, Udaipur, Rajasthan, SNMC, Jodhpur, Rajasthan, KKCTH, Chennai, Tamil Nadu, ICH, Chennai, Tamil Nadu, CMC, Vellore, Tamil Nadu, GVMC, Vellore, Tamil Nadu, Narayani, Vellore, Tamil Nadu, Nalam hospital, Vellore, Tamil Nadu, GRHMMC, Madurai, Tamil Nadu, BCH, Tezpur, Assam, GMC, Guwahati, Assam, KGMU, Lucknow, Uttar Pradesh, Mangla Hospital, Bijnor, Uttar Pradesh, IMS, BHU, Varanasi, Uttar Pradesh, BRDGMC, Gorakhpur, Uttar Pradesh.

Note that full information on the approval of the study protocol must also be provided in the manuscript.

Field-specific reporting

Please select the one below that is the best fit for your research. If you are not sure, read the appropriate sections before making your selection.

☒ Life sciences ☐ Behavioural & social sciences ☐ Ecological, evolutionary & environmental sciences

For a reference copy of the document with all sections, see nature.com/documents/nr-reporting-summary-flat.pdf

Life sciences study design

All studies must disclose on these points even when the disclosure is negative.

Sample size	For the vaccine effectiveness evaluation, the sample size is calculated to achieve 80% power at the 5% significance level to detect OR of 0.6 (vaccine effectiveness of 40%) using a test negative case control study design. The ratio of cases to controls will be 1:2. Expecting the vaccine coverage to be 80% or above for a full series of vaccinations, we would need approximately 242 cases and 484 controls to demonstrate a vaccine effectiveness of ≥40%. To enable further analyses such as genotype-specific vaccine effectiveness calculations, case recruitment will continue throughout the duration of the project irrespective of numbers achieved.
Data exclusions	Based on study design, children who were not eligible for inclusion in vaccine effectiveness analysis were excluded. Any child whose vaccination status could not be confirmed were also excluded.

Replication	The experiments used in the study were independently performed using standardized methods.
Randomization	This was not applicable at recruitment stage as all children under five years of age who were admitted at the study setting for more than 6 hours due to acute gastroenteritis was eligible for enrollment into the study.
Blinding	Blinding was not relevant as enrollment was done at individual sites based on inclusion criteria. As the study was hospital based, screening was done at each setting and eligible children were approached for further procedures.

Reporting for specific materials, systems and methods

We require information from authors about some types of materials, experimental systems and methods used in many studies. Here, indicate whether each material, system or method listed is relevant to your study. If you are not sure if a list item applies to your research, read the appropriate section before selecting a response.

Materials & experimental systems

n/a	Involved in the study
☒	☐ Antibodies
☒	☐ Eukaryotic cell lines
☒	☐ Palaeontology and archaeology
☒	☐ Animals and other organisms
☒	☐ Clinical data
☒	☐ Dual use research of concern
☒	☐ Plants

Methods

n/a	Involved in the study
☒	☐ ChIP-seq
☒	☐ Flow cytometry
☒	☐ MRI-based neuroimaging

Plants

Seed stocks	N/A
Novel plant genotypes	N/A
Authentication	N/A
